# Academy of Medicine Notes

**Published:** 1893-09

**Authors:** 


### Academy of Medicine Notes.

THE programme of the Section on Medicine, it is stated, will surpass anything that has as yet been attempted in Buffalo medical circles. A hearty coöperation with the officers is all that is necessary to insure success.

The next regular meeting of the Academy will be held Tuesday, September 12, 1893, under the auspices of the Section on Medicine.

The annual dues, amounting to $5, are payable in September.

The second year of the Academy should certainly eclipse the first year's record. There is no reason why the Academy should not have a membership of 200 at the close of the year.

The Academy rooms have had a forlorn and deserted appearance during the Summer months. It is expected that this appearance will improve greatly after the sections are in working order.

The Section on Surgery will hold its first Fall meeting Tuesday, September 5th. The programme arranged for the evening consists of a paper on Minor Surgical Operations, by Eugene A. Smith, M. D., and a paper on The Preparation of Suture Material and Dressings, by F. J. Thornbury, M. D.

